# Effect of Natural Aging on Cold Forming Performance of 2219 Aluminum Alloy

**DOI:** 10.3390/ma16093536

**Published:** 2023-05-05

**Authors:** Zhi-Xin Li, Yi-Long Shi, Luo-Peng Xu, Jia-Xin Jin

**Affiliations:** School of Science, Civil Aviation Flight University of China, Guanghan 618307, China

**Keywords:** 2219 aluminum alloy, natural aging, Portevin–Le Chatelier effect, forming performance, microstructure evolution

## Abstract

To facilitate the manufacturing of the thin-walled components of 2219 aluminum alloy, the quenching–forming–aging (Q–F–A) process has been increasingly utilized. However, natural aging (NA) after quenching significantly affects the cold forming performance of this alloy. In this study, experiments are conducted to investigate the effect of NA time on the cold forming performance of 2219 aluminum alloy. The results indicate that NA can weaken the Portevin–Le Chatelier (PLC) effect, thereby reducing its influence on the cold forming performance of the alloy. The PLC effect becomes indistinct when the aging time reaches 2 years. The yield strength of 2219 aluminum alloy increases monotonically with aging time, while the elongation first increases rapidly and then decreases. After an aging time of 2 years, the yield strength increases by 28.6% from that of newly quenched alloys. The strain hardening index and hardening coefficient indicate that short-term NA (less than 4 days) increases the work hardening rate, while long-term NA reduces it. Microstructural analysis shows that the strengthening effect of NA on 2219 aluminum alloy is mainly due to the growth of G.P. zones and the precipitation of θ″ phases. The NA precipitation behavior can also cause the aggregation of solute atoms and weaken the PLC effect.

## 1. Introduction

2219 aluminum alloy is one of the most commonly used alloys for advanced applications, dues to its excellent weldability, good mechanical properties, high specific strength, and good corrosion resistance [1,2,3]. The thin sheets made of this alloy are used to form thin-walled components, which are widely applied in the aerospace, aviation, and automotive industries [4]. Generally, these components are manufactured by cold forming first, then quenching and aging, known as the F–Q–A process. However, in recent years, a high-performance manufacturing process of quenching–forming–aging (Q–F–A) has been developed. This process can not only avoid severe distortion caused by quenching of components but also enhance the mechanical properties of the components further during artificial aging [5,6,7]. Therefore, the alloy undergoes quenching, i.e., solid solution treatment, before the cold forming process. During the waiting period for forming, the as-quenched 2219 aluminum alloy undergoes natural aging (NA). The natural aging process that occurs during waiting can cause changes in both the microstructure and mechanical properties of the alloys, which may have either positive or negative effects on formability. Thus, achieving a proper balance between mechanical properties and formability by controlling the aging time becomes crucial. However, the effect rule and mechanism of NA time (i.e., waiting time) on the cold forming performance of the components are not clear.

For 2219 aluminum alloy, during cold deformation, clear instability can be observed [8]. This instability is evident as repetitive serration in the stress–strain curves, which is known as the Portevin–Le Chatelier (PLC) effect [9]. The PLC effect shows as repetitive serration in the time domain and strain localization in the space domain. This effect causes uneven plastic deformation, negatively affecting material cold forming performance. It results in obvious strip marks on the workpiece surface, greatly affecting both surface quality and service performance [10]. The PLC effect also can lead to localized strain concentrations in forming components and, finally, result in unpredicted failures by imposing loss in their ductility [11,12]. Another study has shown that the PLC effect changes the fracture type and the feature of failure [13]. Thus, it is crucial to implement appropriate measures in forming processes to mitigate or prevent PLC occurrence, enhance ductility and improve forming quality. Currently, Cottrell’s dynamic strain aging (DSA) theory is the main explanation for the PLC effect, which proposes that the interaction between dislocations and solute atoms causes it [14,15]. Excess vacancies may also play a role in this process according to recent investigations [16]. Apart from temperature and strain rate [17,18], the Portevin–Le Chatelier (PLC) effect is mainly influenced by the composition of alloying elements, the solute atom content in the solid solution, and the initial structure. As is well known, the type and concentration of solute atoms in the alloy have an important impact on the dislocation–solute atom interaction, thereby significantly affecting the PLC effect [19,20]. In the study of the effects of precipitated phases, Hu et al. [21] suggest that precipitations have a significant effect on the PLC effect and the greater the amount of precipitation, the more significant the influence for Al-Mg alloys. Moreover, precipitation content could affect the solute diffusion mechanism. Li et al. [22] studied the PLC effect of Al-Li alloys and found that the presence of δ′ precipitated phase promotes the generation of serration and increases the size of serration. The precipitation of the second phase in the aluminum alloy matrix reduces the concentration of solute atoms and weakens their interaction with movable dislocations. This, in turn, affects the PLC effects, including the serration type and critical strain [23,24]. In the NA process, the concentration of solute atoms and the type, morphology, and size of the precipitation in 2219 aluminum alloy will change with aging time. The effect of these changes on the PLC effect of the alloy during the deformation process still remains uncertain up to now. Therefore, it is urgent to explore and comprehend the plastic instability behaviors and mechanisms of 2219 aluminum alloy. In research related to the effect of NA on macroscopic mechanical properties and its microscopic mechanism, Choi et al. [25] investigated the effect of NA on mechanical properties after water quenching for 7075 aluminum alloy, finding that the yield strength increased linearly with the NA time within 2 h. Zheng et al. [26] studied the NA behaviors within 24 h of 7050 (Al-Zn-Mg-Cu) and 5A90 (Al-Mg-Li) aluminum (Al) alloys and found that the initial precipitate (G.P. zones) for 7050 Al alloy had a significantly lower activation energy than the initial precipitate (δ′ phase) for 5A90 Al alloy. Consequently, the NA rate of 7050 Al alloy is more pronounced in the initial stage (after quenching) and decreases dramatically from 0 to 20 h of NA. They recommended an NA time of 20 h. For 2xxx aluminum alloy, Ivanov et al. [27] studied the change of mechanical properties of Al-Cu-based alloys with (Mg, Li) additions during NA, and those results indicated that the NA is finished in 96 h, and then the mechanical strength reaches a stable state. Wu et al. [28] found that the yield strength and elongation of 2195 aluminum alloy rapidly increased and decreased, respectively, within 4 h of aging, and then reached the plateau 24 h later. The major sources of strengthening in the NA state are the δ′/β′ precipitates, G.P. zones, and solute atoms, and they obtained quantitative results of their contribution to mechanical properties. For 2024 aluminum alloy, the aging time required to achieve the plateau is extended to 96 h [29,30].

In terms of the microstructure evolution of 2219 aluminum alloy during the aging process, Papazian [31,32] discovered that aging the alloy at temperatures between 60 °C and 130 °C for a constant mean square diffusion distance of copper results in G.P. zones with an increasing average diameter. The behavior of the dissolution of these zones during DSC analysis can be explained by the effect of zone size on the metastable phase boundary and the kinetics of diffusion-limited dissolution. Wang et al. [33] researched the changes in mechanical properties of 2219 aluminum alloy at various temperatures (150 °C, 165 °C, 175 °C) and discovered that the peak aging time was 24 h at 150 °C to 165 °C, but it reduced to 12 h when the aging temperature increased to 175 °C. Moreover, the microstructure corresponding to the highest strength comprised of the θ′ (Al_2_Cu) phase and a small amount of θ″ phase [7,34]. However, the above studies mainly explored the artificial aging behavior of 2219 aluminum alloy, and the behavior of NA is still unknown. Therefore, it is crucial to investigate and understand the behavior and mechanisms of NA in 2219 aluminum alloy.

This study aims at the dependence of NA time on the cold forming performance and their mechanisms of 2219 aluminum alloy. In tandem with this, the PLC effect, properties, and microstructure evolution of this alloy with various NA time were analyzed first. Then, the effect mechanisms of NA time on the cold forming performance were further examined. The findings of this study can provide theoretical guidance for selecting an appropriate natural aging time in the Q–F–A manufacturing process, thereby improving the cold forming quality.

## 2. Experimental Section

### 2.1. Materials

**Figure 1:**
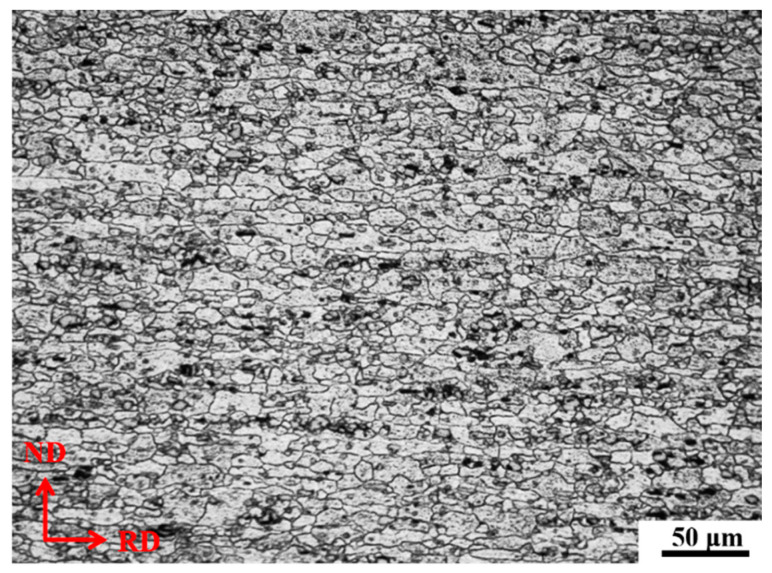
Initial microstructures of cold-rolled 2219 aluminum alloy sheet.

In this study, a 2 mm nominal thickness cold-rolled 2219 aluminum alloy sheet was utilized as the experimental material. The chemical compositions of this alloy in wt% are presented in Table 1. Furthermore, Figure 1 depicts the microstructure in its initial state (as-annealed state), in which RD and ND indicate the rolling direction and normal direction of the rolling plane, respectively. As shown in Figure 1, the initial structure comprises fine equiaxial grains and non-equiaxial grains elongated along the rolling direction. The average grain size measures approximately 20 μm along the rolling direction and 15 μm along the normal direction. The anisotropy of this sheet is visible from this microstructure; however, some research indicates that the impact of aging time on material properties in all directions is comparable [35]. Hence, in our study, research was restricted to the rolling direction alone.

### 2.2. Solution Treatment and Natural Aging

**Figure 2:**
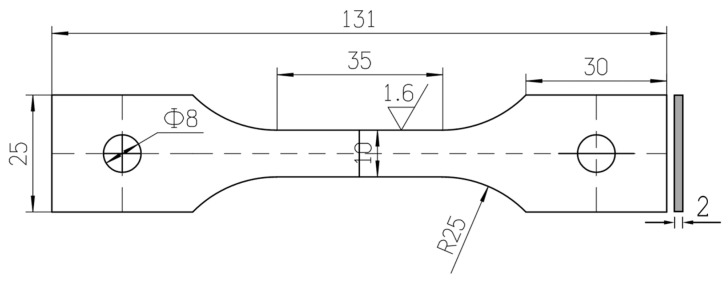
Specimen geometry and size (unit: mm).

The sheet was machined into tensile specimens, and their geometry and size are depicted in Figure 2. Quenching treatment (solution treatment) of the samples was carried out at 530 °C for two hours, followed by water cooling. The initial temperature of water was 25 ± 1 °C. Subsequently, the 2219 aluminum alloy specimens were subjected to different exposure times, namely, 0.2 h, 1 h, 5 h, 10 h, 20 h, 25 h, 96 h (4 days), 240 h (10 days), 1680 h (70 days), 5760 h (8 months), and 17,280 h (2 years). During the natural aging process, the specimens were kept in drying glassware at a temperature range of 10 °C to 30 °C, in ambient conditions.

### 2.3. Material Tests

Uniaxial tensile tests were conducted on specimens with different NA times at room temperature using an INSTRON 5565 material testing machine. A strain rate of 1.2 mm/min was maintained to obtain the load–displacement curves. Each NA time was tested on three specimens. The true stress–true strain curves were derived from these load–displacement curves. The 0.2% offset approach was used to determine the yield stress of the 2219 aluminum alloy.

To examine the microstructures, the FEI Talos F200X transmission electron microscopy (TEM) technique was used. The manufacturer of FEI Talos F200X is Thermo Fisher Scientific, and the company is located in Hillsboro, Oregon, United States. The TEM samples were first mechanically polished and then double-jet electro-chemically polished in a 30% nitric acid and 70% methanol solution. The polishing process was carried out at a temperature of 253 K and a voltage of 20 V.

## 3. Results and Discussion

### 3.1. Analysis of Deformation Behavior

**Figure 3:**
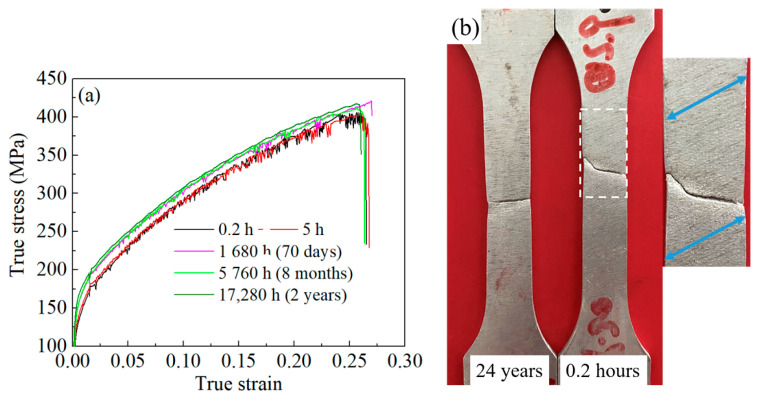
The true stress–strain curves (**a**) and corresponding PLC band characteristics (**b**) of 2219 aluminum alloy with NA for different times.

In Figure 3a, the true stress–strain curves for the as-quenched 2219 alloy with NA for different times are presented. These curves show a serrated stress fluctuation due to the PLC effect. Notably, the serration characteristics differ depending on the NA time, and the serrations have a discontinuous distribution. The degree of serration stress fluctuations decreases as the aging time increases. Figure 3b illustrates the characteristics of PLC bands on the surface of tensile-tested specimens with NA of 0.2 h and 2 years. A comparison of the surface morphology of both specimens reveals that the newly quenched specimen (NA of 0.2 h) exhibits prominent PLC bands, while after aging for 2 years, no significant PLC bands can be observed. Therefore, it can be concluded that the PLC effect of the 2219 aluminum alloy gradually weakens with increasing aging time.

**Figure 4:**
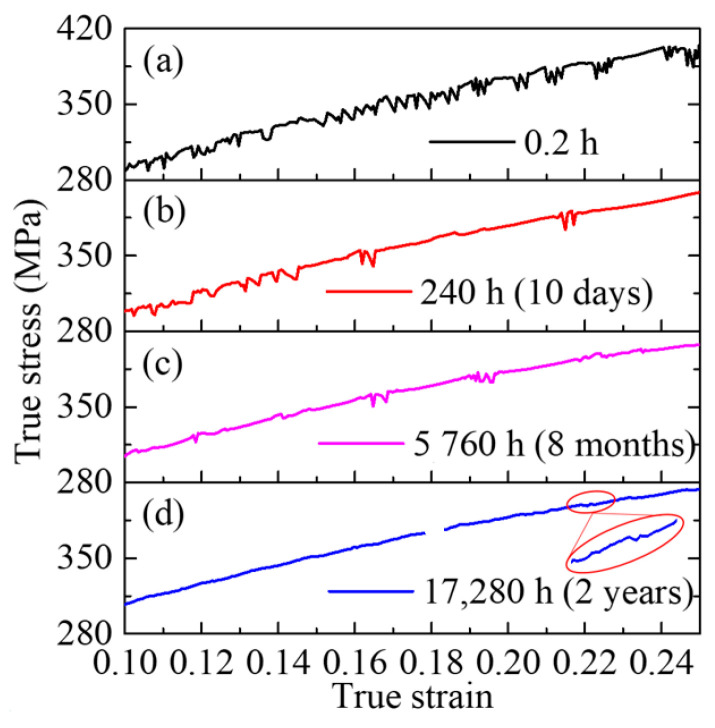
Comparison of the PLC effect in the true stress–strain curves of the alloy after aging for 0.2 h (a), 10 days (b), 8 months (c), and 2 years (d).

Figure 4 displays local magnifications of the true stress–strain curves for the 2219 alloy after aging for 0.2 h, 10 days, 8 months, and 2 years. It can be observed from Figure 4 that the serrations of the true stress–strain curves exhibit different distribution characteristics at different aging times, which is attributed to the PLC effect. The variation in this characteristic with aging time is considered to be that as the aging time increases, the concentration of solute atoms decreases, thereby weakening the role of solute atoms as binding traps for vacancies [11,14]. Previous studies [9,36] have classified PLC effects into three types based on the characteristics of the stress–strain curves, namely, Type A, B, and C, which are characterized by periodic fluctuations, curve oscillations, and curve drops, respectively. When the aging time is less than 8 months, the true stress–strain curves exhibit prominent oscillations, thus indicating that the PLC effect on the alloy belongs to Type B. Additionally, with increasing aging time, the density of serrations and stress drop amplitude gradually decrease. When the aging time reaches 2 years, the serrations become indistinct, demonstrating that the PLC effect is very weak at this stage. According to the dynamic strain aging theory proposed by Cottrell, NA treatment reduces the density of solid solution atoms and vacancies in the aluminum matrix, thereby weakening the pinning effect on movable dislocations in the subsequent deformation process and causing the corresponding PLC effect to be insignificant. Therefore, NA is beneficial in reducing the influence of PLC effect on the cold forming performance of 2219 aluminum alloy and improving the quality of forming.

**Figure 5:**
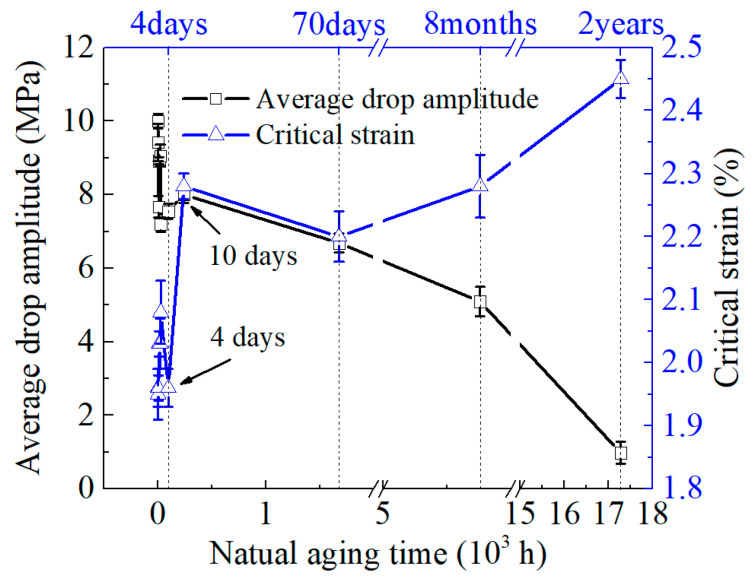
Evolution of average drop amplitude and critical strain of the 2219 aluminum alloys after NA for different times.

In Figure 5, the average stress drop amplitude and critical strain resulting from the PLC effect in the 2219 Al alloy are depicted. The stress drop amplitude refers to the difference between a stress peak and the neighboring stress valley on the stress–strain curve, which reflects the strength of the solute atoms in pinning movable dislocation. The critical strain denotes the strain value at which stress serrations emerge on the stress–strain curve and is tightly associated with atomic diffusion and movable dislocation motion rate.

It is evident that the characteristics of the PLC effect have distinct disparities under different NA time. When the NA time is below 10 days, the average stress drop amplitude is approximately 7–10 MPa and the critical strain is approximately 1.95–2.1%. Once the aging time surpasses 10 days, the average stress drop amplitude shows a declining trend, and it is under 1 MPa when the aging duration amounts to 2 years. Additionally, the critical strain rises rapidly with the advancement of the aging duration, and it is noteworthy that it experiences a sharp increase when the aging time is greater than 4 days.

### 3.2. Evolution of Mechanical Properties

**Figure 6:**
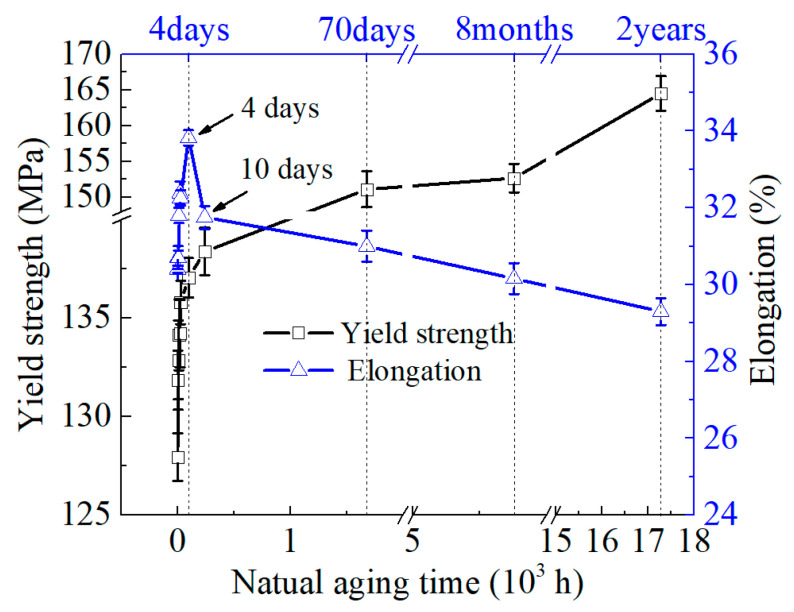
Evolution of yield strength and elongation of the 2219 aluminum alloys after NA.

Figure 6 displays the yield strength and elongation changes of the 2219 aluminum alloy with varying NA time. As shown in the figure, the yield strength of the alloy increases rapidly during the first 4 days of aging, and then the rate of increase gradually decreases with longer NA time. After 4 days of aging, the yield strength of the alloy increases by approximately 7.2%, from 127.9 MPa in the newly quenched state (aged for 0.2 h) to 137.0 MPa. After the maximum aging time of 2 years in this study, the yield strength increases to 164.5 MPa, a 28.6% increase from that of newly quenched alloys. The increase in yield strength will result in a corresponding increase in the forming force during the forming process (such as spinning and stamping) of the alloy. The increase in forming force demands larger equipment tonnage, which results in higher energy consumption.

Regarding the evolution of elongation, it is worth noting that the elongation does not decrease monotonically but first increases and then decreases. As seen in Figure 6, the elongation rapidly increases within the first 4 days of NA and then remarkably decreases within 10 days. Subsequently, the increasing trend slows down as the NA time increases. Moreover, the change in elongation between the alloy aged for 2 years and the newly quenched alloy is not significant, and the maximum elongation occurs at an aging time of approximately 4 days. Indeed, a high elongation at fracture implies a higher forming limit.

In general, aging-strengthening treatment would lead to a decrease in the fracture toughness of some aluminum alloys [26,28]. However, our studies indicate that both the strength and elongation of the 2219 alloy increase rapidly at the early stage when the NA time is less than 4 days. This is probably because that, for quenched metal materials, the quenching residual stress is also a significant factor affecting the fracture toughness of the metal [37], and the quenching residual stress of the aluminum alloy decreases during the early stage of the NA process, resulting in an increase in elongation.

The strain hardening index (*n* value) and hardening coefficient (*K* value) are indicators that demonstrate the work hardening rate of the alloy while undergoing plastic deformation. These parameters hold significant influence on the quality of cold forming process. The maximum level of stress that the alloy starts to neck and the highest achievable uniform strain for the alloy are decided by these two factors. The exponential hardening model, represented by Equation (1), was utilized to determine the strain hardening coefficient and index through curve fitting [38]:

*σ* = *Kε^n^* (1)

**Figure 7:**
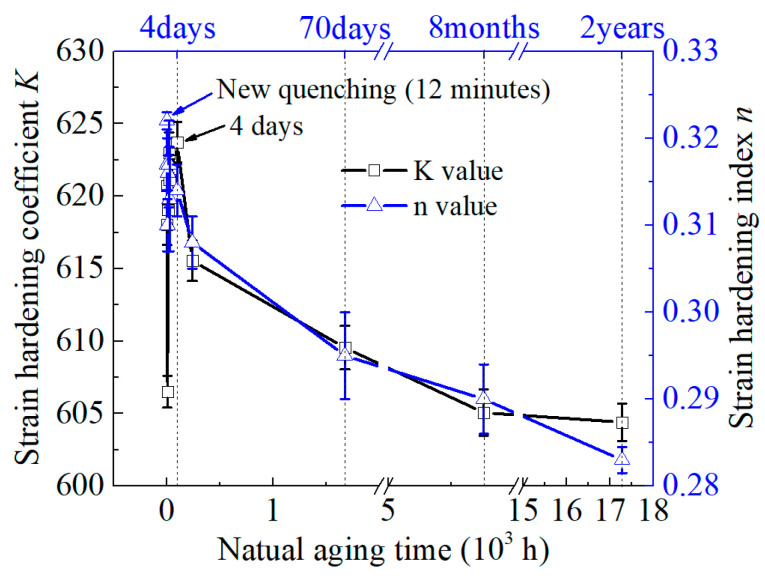
The *K* and *n* values for various natural aging time.

Figure 7 illustrates the changes in the strain hardening index and strain hardening coefficient with aging time for the 2219 aluminum alloy. As depicted in the figure, it is apparent that the newly quenched alloy had the highest value of hardening index *n*, and its value fluctuates around 0.31 during the aging process. However, when the aging time surpassed 4 days, the value of *n* began to steadily decrease. Additionally, as NA progressed, the strain hardening coefficient *K* increased rapidly at first, and then gradually decreased after the aging time exceeded 4 days. The forming process of aluminum alloy sheets with a higher hardening index requires greater forming stress and force and are more likely to experience early fracture. Additionally, a higher hardening index is associated with reduced forming limit of the material. Therefore, long-term natural aging can decrease the work hardening rate and improve the cold forming performance.

### 3.3. Analysis of Microstructures

The precipitation sequence [31,32] of 2219 aluminum alloy is as follows: supersaturated solid solution (sss) → G.P. zones → coherent θ″ → semi-coherent θ′ → stable θ. The precipitation sequence of 2219 aluminum alloy is a complex process that involves the formation of different phases. The first stage, the supersaturated solid solution (sss), is formed when the alloy is rapidly cooled from high temperatures. The next stage involves the formation of G.P. zones, which are regions of the alloy where clusters of solute atoms are segregated. As the aging process continues, these G.P. zones grow in size and transform into coherent θ″ particles. These particles then evolve into semi-coherent θ′ precipitates before finally forming the stable θ phase. The formation of coherent or semi-coherent precipitates creates a robust strain field within the Al-matrix, boosting the resistance of alloy to dislocation motion and thereby affecting its cold forming performance.

**Figure 8:**
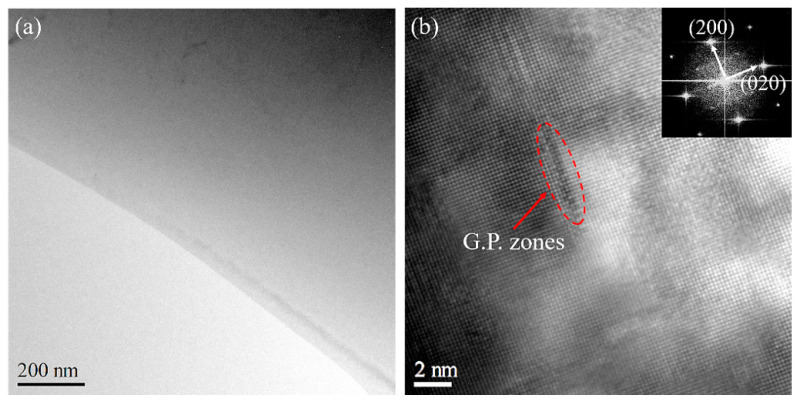
Bright-field TEM (**a**) and HRTEM images (**b**) of 2219 aluminum alloy with aging for 4 days.

Figure 8a,b, respectively, show the bright-field and high-resolution transmission electron microscopy (HRTEM) images of aged 2219 aluminum alloy after 4 days. Figure 8a reveals no apparent precipitation from the bright-field image, while a few G.P. zones with a size of approximately 5 nm are observed in the alloy from the HRTEM image of Figure 8b. This indicates that the concentration of solid solution atoms in the alloy is still high, and the interaction between solute atoms and movable dislocations is still strong. Therefore, it can be seen from Figure 5 that within 4 days of aging time, both the critical strain and average stress drop amplitude maintain a relatively low and high level, respectively, indicating a significant PLC effect. Additionally, in the early stage of aging (less than 4 days), a high-strain hardening index is observed due to the presence of shearable G.P. zones and high solute supersaturation in the alloy.

**Figure 9:**
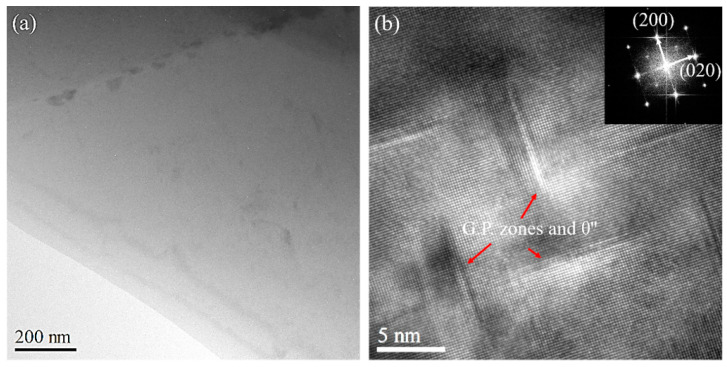
Bright-field TEM (**a**) and HRTEM images (**b**) of 2219 aluminum alloy with aging for 2 years.

Figure 9a shows no observable precipitation phase in the bright-field image after 2 years of aging. However, the number and size of precipitates increase significantly, with larger precipitates growing from 6 nm at 4 days to approximately 10 nm. According to the Selected Area Diffraction (SAD) pattern obtained by HRTEM Fourier transform, these precipitates are G.P. zones and θ″ phases. During the growth of G.P. zones and the formation of θ″ phases, solute atoms aggregate to form solid solution atom clusters. The formation of atom clusters reduces the dispersion density of solute atoms. This causes the time and the minimum dislocation density required for the solid solution atom to catch up with the movable dislocation to increase during the dynamic strain aging process. Thus, the critical strain significantly increases while the average stress drop amplitude decreases when the aging time reaches 2 years, as shown in Figure 5.

The strengthening effect of NA on 2219 aluminum alloy is mainly due to the growth of G.P. zones and the precipitation of θ″ phases. Therefore, the yield strength of 2219 aluminum alloy increases significantly as the aging process proceeds (see Figure 6). However, the increase in the volume fraction of precipitates causes further aggregation of solute atoms such as Cu and Mg. This results in a decrease in the concentration of solute atoms dissolved in the aluminum matrix and weakening of the PLC effect. Therefore, the serrations become inconspicuous when the aging time reaches 2 years, as shown in Figure 4. Moreover, the solute concentration in the alloy rapidly decreases due to the growth of precipitates during NA, and the transformation of G.P. zones θ″ phase, resulting in a decrease in the hardening index (see Figure 7).

## 4. Conclusions

The effects of NA on the cold forming performance of 2219 aluminum alloy were studied through experiments. Based on the obtained results, the following conclusions can be drawn:

(1) The Portevin–Le Chatelier (PLC) effect of 2219 aluminum alloy weakens gradually as the natural aging time increases. The stress drop amplitude of the stress–strain curves is around 7–10 MPa for aging times below 10 days but drops to only about 1 MPa after 2 years of aging. Thus, with an aging time of 2 years, the PLC effect becomes indistinct, indicating that long-term aging can reduce the influence of the PLC effect on the forming process of 2219 aluminum alloy and is favorable for the improvement of cold forming performance.
(2) The yield strength of 2219 aluminum alloy increases rapidly when the natural aging time is less than 4 days, and then the rate of increase gradually decreases. After aging for 2 years, the yield strength of 2219 aluminum alloy increased by 28.6% compared to that of the newly quenched state. However, the elongation first increases rapidly and then decreases, and the maximum elongation occurs at an aging time of approximately 4 days.
(3) The strain hardening index of the 2219 aluminum alloy decreases steadily as the aging time surpasses 4 days. The evolution of strain hardening index shows that short-term natural aging (less than 4 days) increases the work hardening rate of the alloy, while long-term natural aging can reduce the work hardening rate and facilitate stable forming.
(4) The strengthening effect of natural aging on 2219 aluminum alloy is mainly due to the growth of G.P. zones and the precipitation of θ″ phases. The precipitation behavior during natural aging process can also cause the aggregation of solute atoms and weaken the PLC effect.

## Figures and Tables

**Table 1:** Main element composition of 2219 aluminum alloy.

Element	Cu	Mn	Fe	Zr	Ti	Si	Zn	Mg	Al
wt%	6.5	0.36	0.21	0.18	0.06	0.05	0.02	0.01	Remainder

## Data Availability

Not applicable.

## Abbreviations

DSC	Differential scanning calorimetry
F–Q–A	Forming–quenching–aging
NA	Natural aging
HRTEM	High-resolution transmission electron microscopy
PLC	Portevin–Le Chatelier
Q–F–A	Quenching–forming–aging
SAD	Selected Area Diffraction
TEM	Transmission electron microscopy
